# Significant pain decrease in children with non-systemic Juvenile Idiopathic Arthritis treated to target: results over 24 months of follow up

**DOI:** 10.1186/s12969-023-00874-z

**Published:** 2023-08-26

**Authors:** Katinka Spekking, Janneke Anink, Piroska de Boer, Sytske Anne Bergstra, J. Merlijn van den Berg, Dieneke Schonenberg-Meinema, Lisette W. A. van Suijlekom-Smit, Marion A. J. van Rossum, Yvonne Koopman-Keemink, Rebecca ten Cate, Cornelia F. Allaart, Daniëlle M. C. Brinkman, Petra C. E. Hissink Muller

**Affiliations:** 1 Department of Pediatrics, Division of Pediatric Rheumatology, Willem-Alexander Children’s Hospital, Leiden, The Netherlands; 2 De Kinderkliniek, Flevo Hospital, Almere, The Netherlands; 3 Department of Rheumatology, Leiden University Medical Center, Leiden, The Netherlands; 4 Department of Pediatric Immunology, Rheumatology and Infectious Diseases, Emma Children’s Hospital, Amsterdam University Medical Centers, Amsterdam, The Netherlands; 5 Department of Pediatrics/Pediatric Rheumatology, Sophia Children’s Hospital Erasmus Medical Center, Rotterdam, The Netherlands; 6 Department of Pediatrics, Emma Children’s Hospital, Amsterdam University Medical Centers, Amsterdam, The Netherlands; 7 Department of Pediatric Rheumatology, Amsterdam Rheumatology and Immunology Center | Reade, Amsterdam, The Netherlands; 8 Department of Pediatrics, Juliana Children’s Hospital, Hagaziekenhuis, the Hague, The Netherlands

**Keywords:** Juvenile idiopathic arthritis, Treatment-to-target, Treatment strategy study, Pain

## Abstract

**Background:**

The aim of this study was to compare pain-scores in three targeted treatment-strategies in JIA-patients and to identify characteristics predicting persistent pain.

**Methods:**

In the BeSt-for-Kids-study 92 DMARD-naïve JIA-patients were randomized in 3 treatment-strategies: 1) initial sequential DMARD-monotherapy 2) initial methotrexate (MTX)/prednisolone-bridging or 3) initial MTX/etanercept. Potential differences in VAS pain scores (0-100 mm) over time between treatment-strategies were compared using linear mixed models with visits clustered within patients. A multivariable model was used to assess the ability of baseline characteristics to predict the chance of high pain-scores during follow-up.

**Results:**

Pain-scores over time reduced from mean 55.3 (SD 21.7) to 19.5 (SD 25.3) mm after 24 months. On average, pain-scores decreased significantly with β -1.37 mm (95% CI -1.726; -1.022) per month. No significant difference was found between treatment-strategies (interaction term treatment arm*time (months) β (95% CI) arm 1: 0.13 (-0.36; 0.62) and arm 2: 0.37 (-0.12; 0.86) compared to arm 3). Correction for sex and symptom duration yielded similar results. Several baseline characteristics were predictive for pain over time. Higher VAS pain [β 0.44 (95% CI 0.25; 0.65)] and higher active joint count [0.77 (0.19; 1.34)] were predictive of higher pain over time, whereas, low VAS physician [ -0.34 (-0.55; -0.06)], CHQ Physical [ -0.42 (-0.72; -0.11)] and Psychosocial summary Score [ -0.42 (-0.77; -0.06)] were predictive of lower pain.

**Conclusions:**

Treatment-to-target seems effective in pain-reduction in non-systemic JIA-patients irrespective of initial treatment-strategy. Several baseline-predictors for pain over time were found, which could help to identify patients with a high risk for development of chronic pain.

**Trial registration:**

Dutch Trial Registry number 1574.

## Background

Juvenile Idiopathic Arthritis (JIA) is the most common auto-immune disease in children, with an estimated prevalence of 33 per 100.000 children [1, 2]. Pain is a common and distressing symptom of JIA [3, 4]. It can cause sleep disturbances, disrupts school attendance and leads to a decline in quality of life that can persist into adulthood [5–7]. Disease activity is a well-known contributor to pain severity, but not the only contributing factor [3, 8]. In recent years, introduction of (biologic) DMARDs earlier in the disease course has improved the outcome for JIA patients [9–11]. However, this has not directly translated into better pain outcomes as some children experience pain during inactive disease despite adequate treatment and effective disease control [12–14]. A possible explanation for persistent pain with low or no visible disease activity is a decreased pain threshold. Children with JIA were found to have a lower pain threshold than their healthy peers, even when they had no detectable joint inflammation after treatment [15], with longer disease duration as a possible predictor [16, 17]. It has been hypothesized that peripheral and central sensitization, causing prolonged hypersensitivity of pain circuits in the nerve system, contribute to a lower pain threshold [18]. This hypothesis suggests that pain, and the causes of pain should be treated as soon and adequately as possible to prevent sensitization.

In JIA, treatment to target (T2T) with clinical remission as a treatment goal has been widely recommended [19, 20]. Treating JIA to target makes drug-free inactive disease a feasible outcome for an increasing number of children with non-systemic JIA [11, 21]. However, improved patient reported outcomes, such as less fatigue and pain, have not yet been confirmed for treat to target therapy [22].

The aim of this subanalysis is to compare pain scores over two years in three treatment strategies in non-systemic JIA patients who were treated to target aimed at inactive disease [21]. Furthermore, we aim to determine the effect of inactive disease and time to inactive disease on pain in the Best for Kids cohort and to explore and identify baseline characteristics predicting unfavorable pain trajectories.

## Methods

### Patients

The BeSt for Kids study (NTR 1574) is a Dutch multicenter randomized single-blinded trial. It was designed to investigate the effectiveness of three different treat to target strategies for non-systemic JIA patients. Newly diagnosed patients between 2 and 16 years old with JIA (oligoarticular JIA, RF negative polyarticular JIA and juvenile psoriatic arthritis) were included. Exclusion criteria were a disease duration of more than 18 months, uveitis at enrolment and prior DMARD therapy.

Patients were randomized between three strategy arms. Patients in arm 1 were initially treated with methotrexate(MTX) or sulfasalazine(SSZ) monotherapy. Patients in arm 2 initially received MTX and 6 weeks prednisolone bridging. Patients in arm 3 were initially treated with etanercept (ETN) and MTX. Patients were treated to target, with inactive disease (as defined by Wallace [23]) as treatment goal. If inflammation was not enough suppressed and inactive disease was not achieved, treatment was intensified according to the treatment protocol as previously described [21]. Patients were followed every three months until two years. After at least 6 months of inactive disease, treatment was tapered and stopped [21].

The BeSt for Kids study was approved by the Institutional Review Board at Leiden University Medical Center and written informed consent was obtained from all participants before enrolment.

### Outcome measures

The primary outcome measure was pain intensity. This was assessed using a 100 mm visual analogue scale (VAS), where 0 mm is ‘no pain’ and 100 mm is ‘unbearable pain’. Patients were asked to rate their pain over the last 7 days. Under the age of 12, pain was estimated by the parents. A VAS pain of ≤ 35mm is considered ‘mild pain’, whereas ‘moderate pain’ is defined as 36–60 mm and ≥ 61mm as ‘severe pain’ [24].

Inactive disease was defined by the adjusted Wallace criteria [23], where Physician’s Global assessment was measured on a 100 mm VAS and < 10mm indicated no active disease. Baseline number of active joints, Physician Global assessment, VAS patient/parent general wellbeing (0-100mm, where 0 is worst and 100 is best), health related quality of life (HRQoL), symptom duration (time from the first recollection of symptoms) and the use of non-steroid anti-inflammatory drug (NSAID) were tested as possible baseline predictors. HRQoL was measured using the Child Health Questionnaire Parent Form 50 (CHQ-PF50), that includes the physical summary score (PhS) and the psychosocial summary score (PsS) (range 0–100, where 0 is worst and mean (SD) of general US population is 50 (10)) [25, 26]. The number of active joints at each visit was assessed by a physiotherapist or physician who was blinded to the treatment allocation.

### Statistical methods

Descriptive statistics were used with mean and standard deviations (SDs) for continuous variables and absolute frequency percentage for categorical variables. Potential differences in VAS pain scores over time between treatment arms were compared using linear mixed models with random intercept and random slope for visits clustered within patients. The third arm was treated as reference arm since we hypothesized that arm 3 would be superior compared with arm 1 or arm 2, based on earlier research [27]. Possible confounders that were taken into account in this model were sex and age, as some studies suggest these factors might influence pain [28]. This model was performed for the complete 24 months and for the first 3 months separately, as arm 3 proved superior to the other arms concerning disease activity in the first 3 months [29]. A similar multivariable mixed model was used to determine the association between inactive disease and pain intensity, and between time to inactive disease and pain intensity (with all visits except baseline clustered within patients). This model was adjusted for the possible confounders age, sex, treatment group, baseline VAS pain, Physical summary score, Psychosocial summary score, baseline JADAS10 and diagnosis, based on clinical reasoning and previous research [28, 30, 31].

Additionally, a third mixed model was used in an exploratory analysis to assess the ability of several baseline characteristics to predict the chance of high pain levels during follow-up. For this model multiple imputation was used to deal with missing values of variables with missing data from patients who were still in follow-up, as well as data from the 2 patients that were lost to follow-up and performed based on predictive mean matching (with 5 observations to draw from, resulting in 30 imputation sets). Imputation variables were treatment group, age at inclusion, sex, NSAID use, VAS pain, PGA, VAS patient/parent general wellbeing, VAS disease activity, symptom duration at diagnosis, Juvenile Arthritis Disease Activity Score 10 (JADAS10), number of active joints, number of limited joints, Child Health Assessment Questionnaire (CHAQ) score, CHQ- PhS, CHQ-PsS, JIA category, Erythrocyte Sedimentation Rate (ESR), baseline ESR and all baseline outcome measures. We first evaluated the possible predictive variables in a univariable model and subsequently added them to a multivariable model and evaluated its predictive value for reaching a lower VAS pain during follow-up.

For all statistical analyses *p*-value < 0.05 was considered statistically significant. Statistical analyses were performed with SPSS version 25 (SPSS, Chicago, IL., USA) and Stata SE version 16 (StataCorp LP).

## Results

### Patient characteristics

Ninety-four patients were randomized between 2009 and 2014 into the three treatment arms: 32 patients were assigned initial monotherapy (arm 1), 32 patients received initial methotrexate (MTX) with 6 weeks prednisolone bridging therapy (arm 2) and 30 patients initial MTX and etanercept (ETN) (arm 3). Baseline demographics, including proportions of missing data, are summarized in Table 1. Two patients received a revised diagnosis during follow-up and were therefore excluded (one patient in arm 1 and one in arm 3). Two patients who were lost-to-follow-up (one at visit 2, one at visit 5) were included in the analyses.

**Table 1:** Baseline demographic and disease characteristics

	**Arm 1** **Sequential** **Monotherapy** **(*n* = 31)**	**Arm 2** **MTX + 6wks** **Prednisolone** **(*n* = 32)**	**Arm 3** **MTX + ** **Etanercept** **(*n* = 29)**
Age (years), median (IQR)	9.0 (4.7–12.9)	10.2 (6.6–13.9)	8.6 (4.2–12.4)
Symptom duration (mo.), median (IQR)	8.1 (5.5–11.9)	5.9 (4.6–13.3)	8.6 (5.2–13.4)
ANA pos, n (%)	14 (45.2)	11 (34.4)	9 (31.0)
Female, n (%)	23 (74.2)	19 (59.4)	19 (65.5)
**JIA Category**			
Oligo, n(%)	5 (16.1)	3 (9.4)	3 (10.3)
Oligoarticular < 6 months	1	1	3
Oligoarticular > = 6 months	4 (12.9)	2 (6.3)	0
Poly, n (%)	24 (77.4)	25 (78.1)	24 (82.8)
Psoriatic, n (%)	2 (6.4)	4 (12.5)	2 (6.9)
PGA mean (SD) in mm	46.4 ± 15.4	49.7 ± 16.1	51.2 ± 16.6
VAS patient/parent general well-being, mean (SD) in mm	48.9 ± 21.9	56.3 ± 21.4	54.6 ± 22.6
VAS pain mean (SD) in mm	54.7 ± 20.0	54.5 ± 23.0	56.8 ± 22.7
CHAQ, mean (SD)	0.9 ± 0.7	1.1 ± 0.6	1.1 ± 0.5
**CHQ-PF50**			
CHQ-PhS, mean (SD)	32.0 ± 13.4	30.1 ± 12.4	31.0 ± 12.2
CHQ-PsS, mean (SD)	45.4 ± 12.4	46.0 ± 10.5	47.7 ± 9.1
No. active joints, median (IQR)	7.0 (5.0–13.0)	7.5 (6.0–11.8)	8.0 (5.5–13.0)
No. limited joints, median (IQR)	2.0 (0–3.0)	2.0 (1.0–3.8)	3.0 (1.5–5.0)
ESR, median (IQR)	6.0 (2.0–11.0)	6.0 (2.0–23.5)	9.0 (3.5–26.0)
JADAS-10, mean (SD)	16.5 ± 4.2	18.8 ± 4.4	18.8 ± 5.4
NSAID use, n (%)	26 (83.9)	27 (84.4)	25 (86.2)

*MTX* methotrexate, *oligo* oligoarticular JIA, *poly* polyarticular RF-negative JIA, *IQR* interquartile range, *ANA* antinuclear antibodies, *pos* positive, *psoriatic* JIA with psoriasis, *PGA* physician’s global assessment, *VAS* visual analogue scale, *CHAQ* Child Health Assessment Questionnaire, *CHQ-PF50* Child Health Questionnaire parent form 50, *CHQ-PhS* Physical Summary Scale, *CHQ-PsS* Psychosocial Summary Score, *No.* number, *ESR* erythrocyte sedimentation rate, *JADAS-10* juvenile arthritis disease activity score in up to maximum 10 joints. Missing follow-up data occurred in 4% for active joint count, in 4% for limited joint count and PGA, 7% for VAS patient/parent general wellbeing, 1% for VAS pain, 7% for CHAQ score, 14% for CHQ-PhS, 14% for CHQ-PsS and 16% for ESR

### VAS pain

**Fig. 1:**
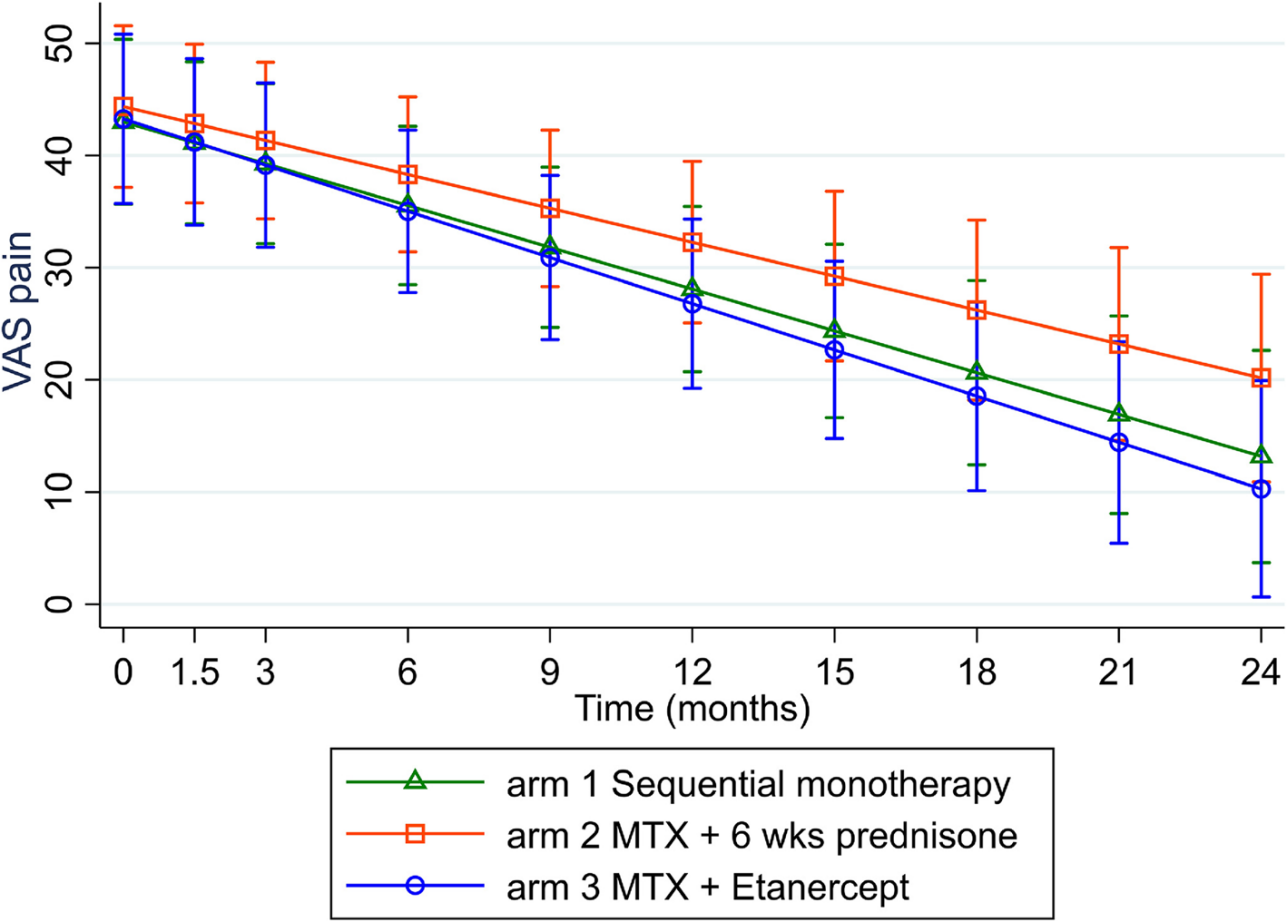
VAS pain outcomes over 24 months in the three treatment arms, based on linear mixed models with random intercept and random slope on unimputed data. Errors bar indicate 95% confidence interval

Mean (SD) pain at 24 months was 17.3 (25.5) mm in arm 1, 25.6 (28.5) mm in arm 2 and 15.8 (21.0) mm in arm 3 (overall 19.5(25.3)). When comparing pain scores over time per treatment arm, pain decreased significantly in every arm with β -1.37 mm (95% CI -1.73;-1.02) per month. No significant difference was found in pain over 24 months between treatment arms (interaction term treatment arm*time (months) in arm 1 with β (95% CI) 0.13 (-0.36; 0.62) and in arm 2 with β 0.37 (-0.12; 0.86) compared to arm 3). Correction for age and sex yielded similar results. Pain trajectories over time are depicted in Fig. 1.

When performing the model for the first three months separately, pain decreased at a similar rate in each treatment group with β(95% CI) -7.82 mm (-11.12; -4.53) per month. Arm 1 compared to arm 3 with β(95% CI) 1.45(-3.14; -6.03) and arm 2 with β(95% CI) 2.33(-2.19; 6.85).

**Fig. 2:**
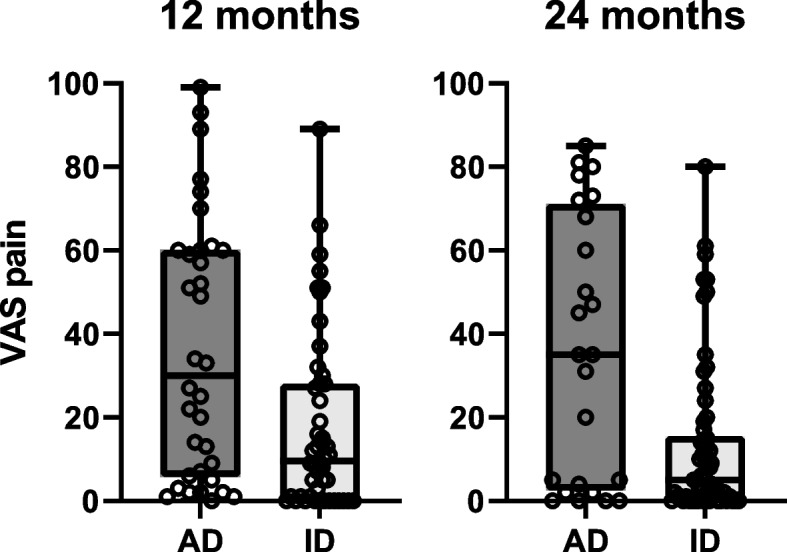
VAS pain active and inactive disease. VAS pain in children with active disease and inactive disease at 12 and 24 months with median and interquartile range. Inactive disease was determined based on adjusted Wallace criteria. At 12 months inactive disease *n* = 48, at 24 months *n* = 65. AD = active disease, ID = inactive disease

Over 24 months, more than 70 percent of patients reached inactive disease [21]. The effect of inactive disease on VAS pain was β -16.87 mm (95% CI -19.65; -14.10) for inactive versus active disease. After adjustment for possible confounders, this effect was β -11.36 mm (95% CI -13.80; -8.93). The effect of time to inactive disease was β 1.33 mm (95% CI 0.60; 2.07) and β 0.52 mm (95% CI 0.11; 0.93) after adjustment for possible confounders. At 24 months, 5 children (8 percent) experienced moderate pain and 2 children (3 percent) experienced severe pain during inactive disease, these seven patients had a mean baseline VAS pain that was 10 mm higher than the overall group. Figure 2 shows VAS pain scores in active and inactive disease at 12 and 24 months.

### Baseline predictors

Several baseline characteristics were selected beforehand and tested for predictive value for pain over time, first in a univariable and subsequently in a multivariable prediction model. In the multivariable model, higher VAS pain and number of active joints at baseline were significantly predictive of higher pain over time, whereas higher VAS physician, CHQ PhS and PsS were predictive of lower pain. Baseline symptom duration, NSAID use and VAS patient/parent were not predictive of pain during follow-up (Table 2). VAS pain showed a β of 0.46 (95%CI 0.25–0.65), indicating that every additional 10 mm VAS pain at baseline is associated with a 4.6 mm higher VAS pain during follow-up.

**Table 2:** Effect of baseline characteristics on pain over time

	**Univariate Analysis**			**Mulitvariate Analysis**		
	**β**	***p*-value**	**95% CI**	**β**	***p*-value**	**95% CI**
VAS Pain (0-100mm)	0.59	< 0.001	0.41; 0.77	0.46	< 0.001	0.25; 0.66
VAS Physician (PGA) (0-100mm)	0.10	0.502	-0.19; 0.40	-0.31	0.013	-0.56; -0.07
VAS patient/parent general wellbeing (0-100mm)	0.36	< 0.001	0.16; 0.57	-0.02	0.831	-0.22; 0.18
Diagnosis						
Poly	2.44	0.744	-12.19; 17.07	-3.84	0.520	-15.51; 7.84
Psoriatic	9.50	0.297	-8.37; 27.38	-2.42	0.750	-17.34; 12.49
No. of active joints	1.20	< 0.001	0.59; 1.81	0.81	0.008	0.21; 1.41
PhS (0–100)	-.0.70	< 0.001	-1.01; -0.38	-0.41	0.011	-0.72; -0.10
PsS (0–100)	-.0.51	0.014	-0.91; -0.10	-0.40	0.036	-0.77; -0.03
Symptom duration (mo.)	8.41	0.147	-3.00; 19.82	8.30	0.117	-2.12; 18.72
NSAID use (yes)	-7.61	0.252	-20.64; 5.41	-0.93	0.858	-11.12; 9.26

Effect of baseline characteristics on pain over time. Included baseline variables in univariate and multivariate analysis; VAS pain, VAS physician, VAS patient/parent general wellbeing, diagnosis, number of active joints, PhS, PsS, symptom duration and NSAID use. *VAS* Visual Analogue Score, *No.* number, *PhS* Physical Summary Score, *PsS *Psychosocial Summary Score, *Mo.* months

## Discussion

The BeSt for Kids is one of the first treat-to-target studies in newly diagnosed, DMARD naïve, non-systemic JIA patients investigating pain as outcome measure. In the current subanalysis, three frequently used initial treatment strategies were compared for effectiveness in treating pain [21, 32]. In the BeSt for Kids study, the treatment target was inactive disease. Pain was not a treatment target, and therefore treatment adjustments were, at least partly, independent of whether patients had residual pain. Nevertheless, our results show that targeted treatment can significantly reduce pain in children with JIA. After 24 months the mean(SD) VAS pain of JIA patients decreased from 55.3(SD 21.7) to 19.5(SD 25.3) mm, at a similar rate irrespective of initial treatment. Pain scores after two years are lower than in earlier studies assessing pain during non-targeted treatment with DMARDs [13, 33]. This suggests that treat-to-target is beneficial in achieving lower pain scores, although direct comparisons are lacking.

Although few studies have addressed pain over time as an outcome, we know from cross sectional data and reports from trials and prospective observational studies that pain scores can remain high after treatment. These high pain levels are reported after treatment according to clinical practice before the availability of biologic treatment [34, 35], but also in patients in whom disease activity has declined after successful treatment with abatacept and other biologicals [3, 13, 33]. Even in the biologic era, pain can remain present in the long term, and high pain perception affects quality of life [12, 14]. The decrease in pain seen in patients participating in our treat to target strategy is therefore promising.

Even though we saw a decrease of active disease and pain in the BeSt for kids study, still some of our patients who did achieve inactive disease continued to have pain. It is this group of patients that deserves additional attention [36, 37]. Central sensitization could be a factor involved in persistence of pain in chronic conditions like JIA [38]. Our analyses showed that longer time to inactive disease has a small, but significant association with more pain over time. Further research should focus on how to identify patients who are at risk for chronic pain even after the inflammation is abrogated. When we are able to recognize children with chronic pain in an early stage, they might benefit from education about the cause of their pain, relevant pain mechanisms, and the role of psychosocial and physical factors in precipitating and maintaining chronic pain [39, 40].

In addition to this, we could consider adding an extra patient reported outcome, to investigate whether the clinical improvement we find corresponds to what our patients regard as a satisfactory improvement in pain levels [37, 41, 42]. In adult RA it is recognized that clinicians may have another perspective on how a favorable outcome is defined than their patients, and research is increasingly directed towards more patient reported measures and a value based perception of health care [43, 44]. Recently, in adult rheumatology, the dual target strategy was proposed to enhance patient satisfaction, with one target representing control of inflammation (biological remission) and the other control of disease impact (symptom remission) [45].

We performed an exploratory analysis to identify baseline factors that predict pain over time. In univariable analyses we identified VAS pain, PGA, VAS patient/parent, number of active joints, PhS and PsS as significant predictors of pain over time. In a multivariable model including all previously tested variables, identified VAS pain, PGA, number of active joints, PhS and PsS remained predictive. This is in accordance with previous literature [31, 46–48].

Counterintuitively and in contrast to previous studies, in our multivariable model, a lower PGA at baseline was associated with more pain during the two years follow up [8, 46]. This reversal of the effect compared to the results of our univariable analyses is related to the combination of predictors in our multivariable model. Due to the predictive nature of our model, this relationship with pain should not be interpreted causally.

Contrary to previous studies we did not find symptom duration as a significant baseline predictor for more pain over time. This could be due to lack of power, as these studies included more patients and the effect that was found was very small [8, 46].

In our previous publication [21], we reported intraarticular corticosteroid injections that were administered outside the study protocol. This happened rarely, but more frequently in arm 2 than in other treatment arms. Despite this difference in frequency, we do not see a difference in pain course over time, which could be attributed to the intraarticular injections.

There are some limitations to our study. The sample size of our study was limited, therefore more subtle differences might have been found in a larger study cohort. Second, pain is multifactorial in nature and affected by many psychosocial patient factors [39]. This study only looked at pain intensity and did not take a possible response shift during the study period into consideration [49]. Third, VAS pain scores were completed by patients or their parents. Parents were encouraged to obtain self-report by the child, but in young children this was not always possible. Patient-proxy reports are known to not always correspond, especially in children with a high disease burden [50]. Fourth, pain can be influenced by sensitization [18, 38]. This might have occurred prior to start of treatment. In our analyses, we have only looked at time to start of treatment, but other factors may have influenced sensitization and pain. And lastly, the prediction model was based upon a randomized cohort with strict inclusion criteria. To participate in the BeSt for kids study, symptom duration had to be less than 18 months. This and other inclusion criteria might limit the generalizability of the results.

## Conclusions

We conclude that treat to target therapy is effective in reducing pain over time for DMARD-naïve non-systemic JIA patients, irrespective of initial treatment. On the other hand, some children still experience pain despite achieving clinically inactive disease. This emphasizes the necessity of addressing patient related outcomes in addition to targeted treatment to reduce disease activity. Several baseline predictors for pain over time were found, which could help to identify and support non-systemic JIA-patients with a high risk of pain in addition to a treat-to-target strategy treatment.

## Data Availability

The datasets used and/or analyzed during the current study are available from the corresponding author on reasonable request.
